# Interface Reinforcement of Pulp Fiber Based ABS Composite with Hydrogen Bonding Initiated Interlinked Structure via Alkaline Oxidation and *tert*-Butyl Grafting on Cellulose

**DOI:** 10.3390/polym11122048

**Published:** 2019-12-10

**Authors:** Qinrui Zhu, Dagang Li

**Affiliations:** College of Material Science and Engineering, Nanjing Forestry University, No. 159, Long Pan Road, Nanjing 210037, China; zhuqinrui1996@njfu.edu.cn

**Keywords:** ABS, modification, interfacial adhesion, compatibility, interlinked structure

## Abstract

Interface optimization in preparing natural fiber based biocomposite becomes a key factor that determines overall properties, especially mechanical performance. The solution for upgrading interfacial adhesion stemmed from polar fiber and nonpolar polymer remains unclear. Here, a kind of pulp fiber/acrylonitrile-butadiene-styrene (ABS) composite with content ratio of 1:1 was fabricated by functionalizing the cellulose fiber to coordinate interaction between fiber and ABS. With addition of 5 wt % polyacrylamide (PAM) there existed an interlinked three-element structure in composite. Three types of treatment to cellulose fiber, including alkali immersion, pivaloyl chloride grafting for 10 h and 20 h were conducted. Pulp fiber that was treated with alkali for one hour, followed by pivaloyl chloride reaction for ten hours, proved to be effective for interfacial adhesion. X-ray Photoelectron Spectroscopy (XPS) analysis reveals 21.9% of carbonyl and 12.1% of ester function in this fiber, which corresponds to oxidation and grafting. For its composite SEM picture displays that most of cellulose fiber are rooted in ABS and evident traces of tearing or fracture can be observed after tension test. DMA test indicates that this modified pulp fiber/ABS composite exhibits great compatibility, because of combined loss modulus peak ranging from 80 °C to 100 °C. Moreover, the well miscible composite has a tensile strength of 58.1 MPa and elastic modulus of 2515 MPa, increasing by nearly 50% and 60% from those of pure ABS, respectively.

## 1. Introduction

Acrylonitrile-butadiene-styrene (ABS) synthesized from acrylonitrile, butadiene, and styrene is a wide-used engineering polymer. It is well known for anti-chemical erosion, excellent flexibility, and appropriate rigidity. As a kind of thermoplastic material, ABS can be molded into various shapes or compounded with other polymers for further application. For instance, ABS/PC composite that was prepared by twin screw extruder is considered as crust of electronic device for impact resistance and insulation. PA added in ABS increases the tenacity of composite and in automobile ABS/PA is manufactured into panels. Most countries have replaced general plastics, such as polypropylene and polyvinyl chloride for glass fiber reinforced ABS in construction industry, as well because of its outstanding flexural strength. To some extent, the blending of ABS and other polymers expands the application of ABS in packaging industry.

Although ABS composites have been extensively popularized in many sections, some drawbacks also exist. Those petroleum-based plastics produces much pollution during raw synthesis. In melt compounding, high temperature and pressure are applied, which is labelled as an energy intensive process. More importantly, these non-degradable composites bring about obstacles in recycling and violate the principle of environmentally friendly development. By contrast, when comparing to traditional polymers, natural fiber derived from plants and trees consists of cellulose, hemicellulose, and little glucide. A great aspect ratio of cellulose fiber, together with high crystallinity induced by hydrogen bonding, renders supreme mechanical properties. As a reinforcing filler, natural fiber has light weight and low processing temperature. Its blending with ABS provides degradability for composite. In turn, ABS makes up the shortage of chemical resistance of natural fiber. It seems that natural fiber reinforcing ABS has the potential to take place of conventional ABS composites.

Natural fiber/ABS composites possess poor compatibility between polar fiber and nonpolar ABS when compared to composites that were constituted by several nonpolar resins. The research core of natural fiber reinforcing ABS is to optimize interface by manipulating the interaction of different molecules in blenders, so as to increase the miscibility. It means that in the manner of adjusting the type or distribution of characteristic groups phase interface can be converted from discrete state to successive interwoven state. Two strategies, including modification of cellulose and addition of compatibilizers, are adopted to consolidate interface. Yano et al. [1] reported cellulose fiber that was modified by alkenyl succinic anhydride (ASA) had positive effects on the reinforcement of HDPE. It was concluded that pulp fiber with degree of substitution (DS) of 0.43 dispersed well in the matrix and the tensile strength of this composite increased by 143%. Other methods that were focused on functionalized grafting of cellulose with small molecules were widely spread in preparing biocomposites [2,3,4]. Meanwhile, Akato et al. [5] found that the ABS composite containing 30 wt % lignin exhibited great interfacial adhesion with the assistance of polyethylene oxide (PEO). Similarly, graphene oxide sheets were also used as compatibilizer for immiscible system [6,7]. This implies the introduction of special groups or bonding activates interaction among the components in composite.

However, it is noticed that the efficiency of chemical modification is limited and the promotion of compatibility can only be realized in composite with low filling fraction of natural fiber. The high filling of natural fiber in plastics is seldom reported for difficulty in optimizing interfaces and related poor mechanical properties. There is no denying that composites incorporating a high content of natural fiber enlarge the advantages of biomaterial, such as easy degradability, green sustainability, and low carbon emission. We investigated interfacial improvement of ABS/pulp fiber composite at 1:1 ratio in order to prepare ABS composite with high filling natural fiber.

In this article, the modification on pulp fiber was carried out for accommodating the features of ABS. Research in effects of conditions of chemical treatment upon functional groups of cellulose fiber was conducted based on the molecular structure of cellulose. PAM was added in the blender to stabilize the compounding system. Subsequently, compatibility between modified fiber and ABS was evaluated for exploring potential mechanism of interface consolidation. The critical technique for improving miscibility was to functionalize cellulose fiber. By oxidating and esterifying cellulose the changed functional groups triggered hydrogen bonding among polymers, thus strengthening the interfacial adhesion of phases. The major concern of research was linkage of oxidation and esterification level to the properties of composite. Distinguished from previous modification [8], our process included alkali immersion for swelling up cellulose fiber [9], prior to esterification and reduced dosage of reagent for catering to green chemistry. The efficiency of treatment following two steps was discussed. Through compounding via twin screw extruder a three-element reinforcing structure that was composed of modified cellulose fiber, PAM, and ABS had been built in situ.

## 2. Experimental Section

### 2.1. Materials

ABS (trade name: HI100) with melt flow rate of 11 g/10 min. under load of 10 kg at 220 °C (ASTM D1238) was provided by LG Chem Corporation (Seoul, South Korea). Its tensile strength and tensile elongation at yield in 23 °C were 37 MPa and 5%, respectively (ASTM D638). Yang Run trade limited corporation (Da Lian, China) supplied pulp fiber that was derived from needle leave wood. Water content of pulp was lower than 10%. Pivaloyl chloride (T109597-100 mL) with purity of 98% was bought from Aladdin (Shang Hai, China). Ya Tai Union Chemistry Co. Ltd produced *N*-methyl pyrrolidone (NMP) that was used as solvent with purity above 99% (Wu Xi, China). Pyridine (P111513-100 mL) with purity of 99.8% was purchased from Aladdin (Shang Hai, China) and used as catalyst. Anionic polyacrylamide (APAM) with molecular weight between three-million and 25-million and solid content above 90% was obtained from Teng Long water purification corporation (Zheng Zhou, China).

### 2.2. Sample Preparation

Dry pulp was put into distilled water for preparing 2 wt % suspension. Subsequently, 16 wt % sodium hydroxide solution was formulated and poured in suspension. Before stirring the mixed suspension temperature remained constant at 40 °C. Alkali treatment was performed for about one hour. After immersion in alkali solution, pulp fiber was washed by distilled water and dried in vacuum at 60 °C. Subsequently, the dried fiber was picked in *N*-methyl pyrrolidone (NMP) solvent followed by continuous stirring. Prior to adding pyridine and pivaloyl chloride in NMP suspension temperature of system was raised to 90–100 °C for the removal of residue water in the case that pivaloyl chloride reacted with water. At the end of reaction pulp fiber was filtered and washed by glacial acetic acid, ethanol, and distilled water [10]. ABS and PAM were conducted at content ratio of 1:1:0.1 and the blender was grinded by three-roll grinder in order to complete defibrillation premixing of modified fiber (S65 Ying Zhi machine Co. Ltd, Chang Zhou, China). Afterwards, the mixed powder was melting compounded at 170 °C with screw rate of 35 rpm via twin screw extruder (HAAKE MiniLabⅡ, Thermo Fisher, Karlsruhe, Germany). Afterwards, small bars of composite from extrusion were processed into dumb bell shaped samples at 185 and 110 °C for melting and molding, respectively, under pressure of 6–8 kPa by micro injection molding machine (WZS 10D Xin Shuo equipment Co. Ltd, Shang Hai, China).

### 2.3. Characterization

Tension Test: A sample in shape of dumb bell with size of 25.4 mm × 3.5 mm × 3.2 mm in length, width, and height was obtained from injection molding. According to method of ASTM D638 V tensile clamp with BSS-100 kg AT and 2.9998 mv/V was selected in tension test by a multi-functional mechanical test machine (CMT4204 Sans Testing Machine Co. Ltd, Shen Zhen, China). Each kind of sample was tested five times. Stress-strain curve of each sample was plotted after testing.

Scanning Electron Microscopy (SEM): The cross section of fractured dumb-bell shaped samples was observed after the tensile test. Cross section had been gold-sputtered and the surface morphology was investigated by SEM instrument (Quanta 200 FEI, Beaverton, OR, USA), with an acceleration voltage of 20 kV.

Fourier Transform Infrared Spectroscopy (FTIR): Different types of chemical groups on cellulose fiber under specific treatment were analysed. The FTIR spectra of the modified and unmodified cellulose were collected over the wavenumber range of 4000–600 cm^−1^ at resolution of 3 cm^−1^ by Nicolet IS10 spectrometer (Thermo Fisher Scientific Inc, Waltham, MA, USA). Each kind of sample was observed four times. The changes of peaks attached to specific groups were identified.

X-ray Photoelectron Spectroscopy (XPS): XPS can classify the main elements of cellulose fiber and characterize the types of bonds corresponding to main elements in detail. The C1s accurate spectra of modified and unmodified cellulose fiber were obtained over the binding energy range of 278–296 eV via AXIS UltraDLD instrument (Shimadzu, Kyoto, Japan) equipped with a 165 mm hemispherical electron energy analyser and a monochromatized Al Kα (600W) source. Analysis was done with a depth of 2–3 nm.

Dynamic Mechanical Analysis (DMA): DMA was used to discuss the changing process of storage modulus (E’) and loss modulus (E’’) of composite samples, so as to compare the softening points (*T*_g_) and compatibility between cellulose fiber and ABS. Samples measuring 16 mm × 4.5 mm × 3.5 mm were tested with maximum dynamic force of 4N and heated from 0 to 150 °C at a rate of 3 °C/min. The DMA test was carried out in single cantilever mode with an amplitude of 50 μm and frequency of 1,2,5 Hz via DMA 242D instrument (Netzsch, Selb, Germany). Each kind of sample was observed five times.

Addition in sample preparation: Two types of chemical modification for pulp fiber differing in content of reagents and reaction time were listed in Table 1 below. As a reference item, the procedure without modification was also recorded.

Type1 only included alkali treatment for pulp. It mainly intended to remove hemicellulose and impurity. Type2 added alkali treated pulp into NMP solvent and put enough pyridine as catalyst with continuous stirring. Based on theoretical calculation for preparing modified cellulose with degree of substitution (DS) 0.4, which was reported as an optimal trial according to comparative discussion and substitution of alkenyl succinic anhydride (ASA) by Yano et al. [1], addition of pivaloyl chloride referred to mass ratio of pivaloyl chloride and pulp fiber at 1:1.6. The whole system remained for 10 h at 80 °C in stirring condition. When compared to type2, type3 reduced the amount of catalyst, but added more pivaloyl chloride and then increased the reaction time to 20 h. By designing different reaction conditions modification with longer reaction time and more volume of reactant was contrasted with that including a shorter time, but more capacity of catalysis. Unlike Agustin’s method, which adopts acyl chloride to hydroxyl molar ratio of 5:1 with excessive pyridine [8], this procedure generally reduces the volume of reagent and investigates synergistic effects of alkali treatment and esterification on cellulose.

## 3. Results and Discussion

In this part Type1, Type2, and Type3 mentioned before were abbreviated to T1, T2, and T3. We denoted those pulp fibers that were treated by T1, T2, and T3 as pulp1(P1), P2, and P3, respectively. Similarly, we denoted those composite samples containing P1, P2, and P3 as sample1(S1), S2, and S3.

### 3.1. Tensile Properties

**Figure 1:**
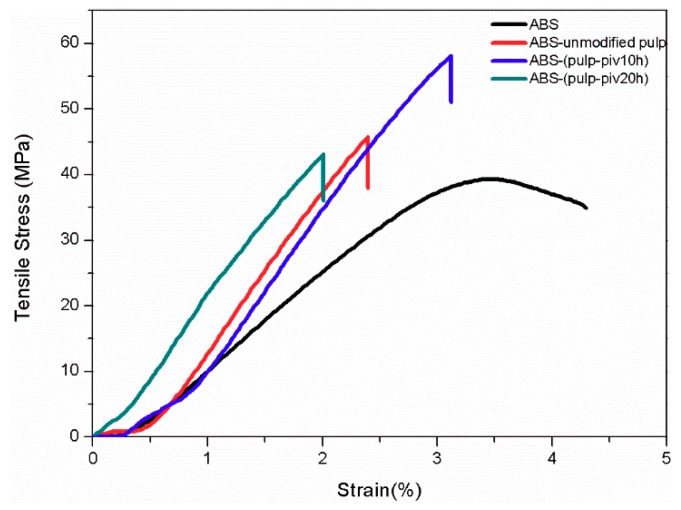
Stress-Strain curves of pure ABS and pulp fiber/ABS composites. (“pulp-piv10h”, “pulp-piv20h” represent T2 and T3 modified pulp fiber. “unmodified pulp” is only alkali treated pulp fiber (T1 treated pulp fiber)).

The mechanical performance of three sorts of composite samples was displayed with that of pure ABS in Figure 1. Table 2 lists their tensile strength and tensile modulus.

From the Stress-Strain curve, S2 exhibited the highest tensile strength (58.1 MPa). When compared to pure ABS, the tensile strength of S2 almost increased by 50%. While S1 and S3 had similar tensile strength, 45.8 and 43.1 MPa, respectively. They raised slightly from 39.3 MPa of pure sample. When it comes to tensile modulus, it can be seen that pure ABS had lower elastic modulus than any other because before reaching the yielding point the tensile stress of pure ABS did not increase as sharply by strain as others. S3, with the highest elastic modulus of 2640 MPa, presented rigidity in the tensile test. Slightly decreasing from the modulus of S3, the elastic modulus of S1, S2 remained the same level of 2567 and 2515 MPa. Moreover, apart from pure ABS whose curve had a yielding stage and showed characteristics of ductile fracture, the rupture performance of other samples exhibited brittleness owing to stiff natural fiber filling in ABS. All three kinds of composites improved their tensile strength despite inflexibility brought about by high-filling cellulose fiber. However, the increasing in tensile strength differed among these composites. It was mainly attributed to distinction in interfacial adhesion between fiber and ABS, which was vital to load transfer [11].

### 3.2. SEM Observation

SEM observation for surface morphology of fracture cross section was conducted to verify this assumption.

**Figure 2:**
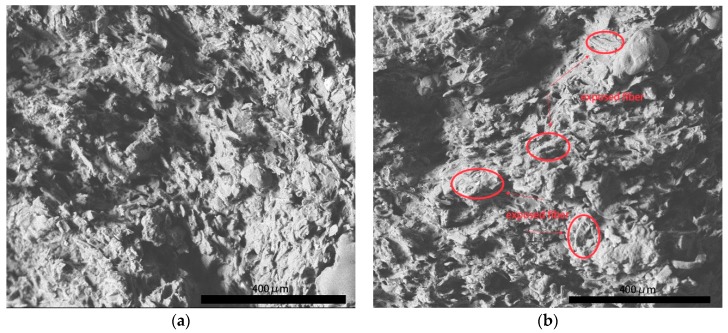
Cross section of S1 after loading with tensile stress. (Note. On the (**a**) is general fracture morphology of S1 at a scale bar of 400 μm. On the (**b**) is featured fracture morphology of S1 at a scale bar of 400 μm).

**Figure 3:**
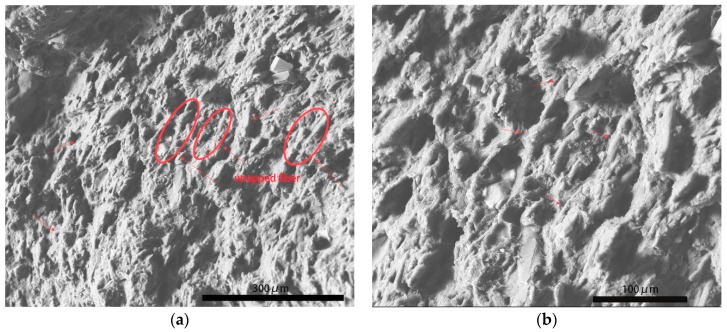
Cross section of S3 after loading with tensile stress (on the (**a**) is fracture morphology of S3 at a scale bar of 300 μm. On the(**b**) is enlarged morphology in red circles at a scale bar of 100 μm).

**Figure 4:**
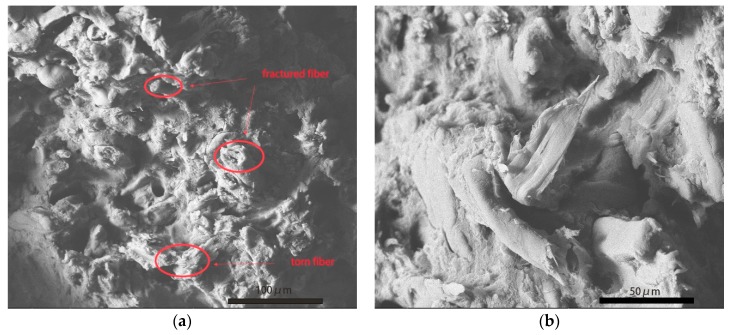
Cross section of S2 after loading with tensile stress. (On the (**a**) is fracture morphology of S2 at a scale bar of 100 μm. On the (**b**) is fracture morphology of S2 at a scale bar of 50 μm).

As shown in Figure 2a, there were many dark circles that were distributed in the bright section. They represented decentralized rubber phase was dispersed in continuous plastic phase, which was a characteristic of ABS [5,12]. The rough surface indicated natural damage by tensile stress. Some thin strips with length about 40 μm were found laid on the ABS in a certain direction along inclined 45 degrees of picture plane. They were pulp fiber extracted under tensile stress. This regular orientation of pulp fiber depended on the shear force from twin screw extrusion. In Figure 2b, shapes of pulp fiber can be clearly seen. Signed by red arrows, cellulose fiber in S1 was exposed on the surface of plastic phase. There was no significant damage to fiber after loading with tensile stress. It meant poor interfacial adhesion between fiber and ABS, resulting in easy ‘pull out’ of fiber by tensile stress [13]. Without firm adhesion and great load transfer via filled fiber mechanical reinforcement can not be highlighted in S1. Similar to fiber of S1, those fiber that were included in S3 also appeared parallel to the cross section, as shown in Figure 3a. The size distribution of them in diameter was more even than that in S1. Specifically, P2 and P3 are well fibrillated. Pulp fiber in S3 was found to be shorter in diameter, because T3 modification led to a slight degradation in cellulose fiber (referring to discussion about FTIR and XPS present below). Whereas, it can be seen that fiber of S3 was partly enclosed by ABS without being completely pulled out. Marked by red circles in Figure 3a, the middle of pulp fiber was uncovered with residue resin and the ends of them were imbedded in matrix. Enlarged morphology in Figure 3b reveals after tensile damage there still existed wrapped fiber rather than totally exposed fiber. This phenomenon proved that the T3 modified pulp fiber had better interfacial adhesion with ABS. Furthermore, different from P1 and P3, T2 modified pulp fiber was firmly inserted in ABS after fracture, as illustrated by Figure 4a. These fiber maintained upright orientation and were coated with ABS inside composite. The fracture surface of fiber was obviously seen with irregular serration, which was an indicator of bearing tensile load. From Figure 4b, there also existed evident traces of tearing on the top of pulp fiber. Despite the damage to fiber under tensile stress, none of them were separated from ABS. In contrast, great interfacial adhesion ensured that enveloped fiber was anchored in ABS matrix. All of these demonstrated strong interaction at the interface of fiber and ABS. Benefiting from T2 modification, P2 exhibited better interfacial miscibility with ABS than that of P1 and P3. When loaded with tensile stress these modified fiber played an important role in transferring stress and preventing concentric stress from damaging vulnerable defects in composite. In brief, modified natural fiber firmly rooted in matrix can enhance mechanical properties of composite due to improved interfacial adhesion.

### 3.3. FTIR Analysis

**Figure 5:**
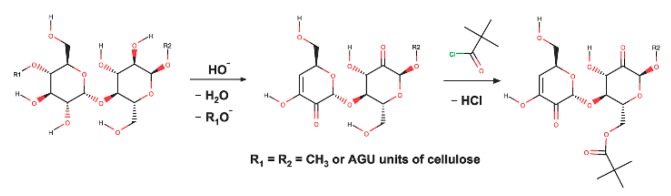
Scheme of changed functional groups on cellulose.

**Figure 6:**
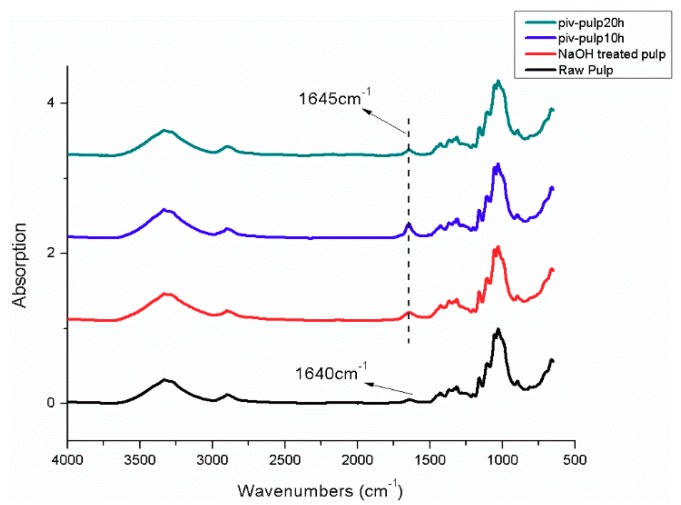
General Fourier Transform Infrared Spectroscopy (FTIR) spectra of P1, P2, P3, and raw pulp fiber (“piv-pulp10h”, “piv-pulp20h” were abbreviation of 10-h pivaloyl chloride modified pulp fiber (P2) and 20-h pivaloyl chloride modified pulp fiber (P3).).

Functionalized cellulose was prepared and three types of treatment for cellulose fiber were investigated in order to strengthen interfacial adhesion. Figure 5 shows the scheme of changes of functional groups on cellulose. The normalized FTIR spectra that are shown in Figure 6 analysed sorts of chemical groups of raw pulp fiber, unmodified cellulose fiber (P1, only alkali treated), 10-h pivaloyl chloride modified fiber (P2), and 20-h pivaloyl chloride modified fiber (P3).

**Figure 7:**
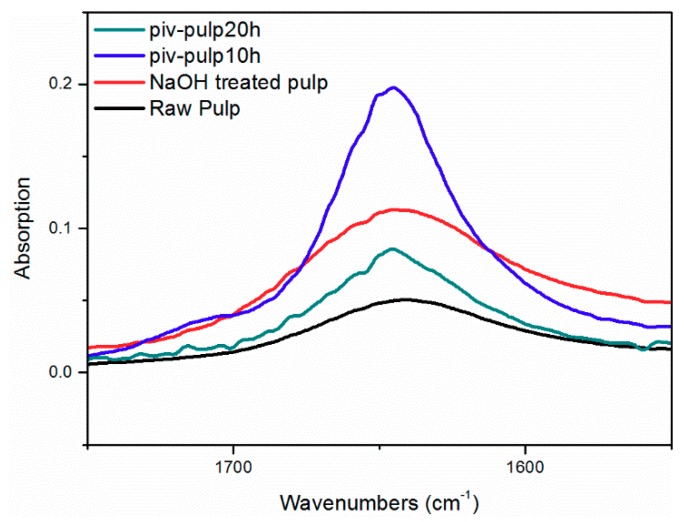
Enlarged FTIR spectra ranging from 1600 to 1700 cm^−1^.

In Figure 6, a broad peak between 3500–3300 cm^−1^ with similar absorption intensity among four spectra is attached to hydrogen bonds and free hydroxyl groups on cellulose chain. The peak at 2898 cm^−1^ is related to stretch vibrations of CH. For a wavelength below 1500 cm^−1^, the band around 1000 cm^−1^ is attributed to COC (1160 cm^−1^) and coexistence of peaks at 1052 cm^−1^, 1104 cm^−1^ is evidence of primary and secondary alcohol [14,15,16]. They are part of the backbone structure of cellulose. It is noticed that in the spectrum of raw pulp there is a weak absorption peak at 1640 cm^−1^, while this peak is intensified and it appears at 1645 cm^−1^ in the other three spectra. Associating carbonyl groups existing in a small amount of hemicellulose or adsorbed water might be responsible for the peak at 1640 cm^−1^ [17]. After alkali treatment and addition of pivaloyl chloride oxidation process in cellulose converts hydroxyl groups into carbonyl [18]. This process is followed by formation of olefinic bonds that lead to conjugation and degradation due to β-alkoxyl elimination [19]. As a result, the amount of carbonyl increases and reduced association via hydrogen bonding renders unsaturated carbonyl to shift toward 1645 cm^−1^ [20]. Different absorption intensity of peaks at 1645 cm^−1^ are connected with degree of conjugate action. The lowest peak intensity of P3 implies a high level of conjugation and, hence, more cleavage of cellulose chain as guided by Hosoya’s model [19]. This can result from relatively intense alkaline oxidation (confirmed by XPS). However, a tiny peak around 1720 cm^−1^ is indistinctively detected in spectrum of P2, as shown by Figure 7. This peak corresponds to C=O vibrations from the ester functions generated by esterification with pivaloyl chloride [21]. What contributes to the uncommon performance might be that prior alkali treatment renders hydroxyl to adsorb hydrated sodium hydroxide, which reduces the amount of free hydroxyl groups in cellulose and, thus, weakens the reactivity with pivaloyl chloride [17,18]. It can also not be neglected that reagent with tertiary butyl, like pivaloyl chloride, has the nature of limited degree of substitution on cellulose. Yano’s group found the maximum DS of 0.6 [8]. When extending reaction time to 20 h, one can hardly find signal for ester. The desorption of incorporated alkali under longer time provides expedient alkaline condition for oxidation on cellulose, from which delocalization derives and inhibits further esterification. A recent study concerning oxidizing systems and degradation model of cellulose has already been reported [22]. Actually, this controllable oxidation combined with minute grafting on cellulose optimizes the interfacial adhesion of composite. Specific mechanism and evidence regarding phase interaction can be found in the DMA section.

### 3.4. XPS Analysis

**Figure 8:**
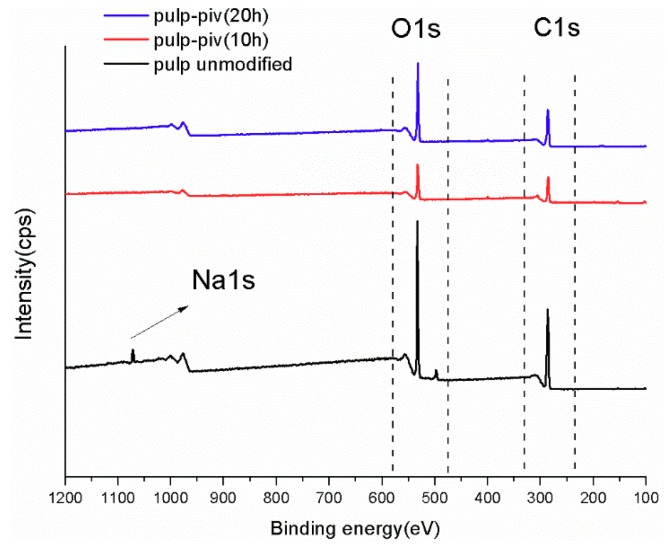
X-ray Photoelectron Spectroscopy (XPS) full spectra of P1, P2, and P3 (“pulp unmodified“ represents P1 that was alkali treated only. “pulp-piv(10h)”, “pulp-piv(20h)“ represent P2 and P3, respectively.

**Figure 9:**
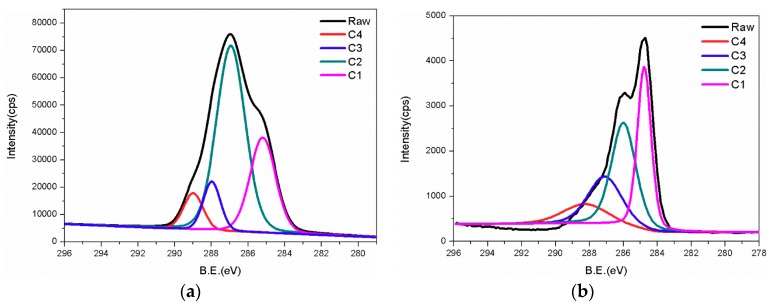

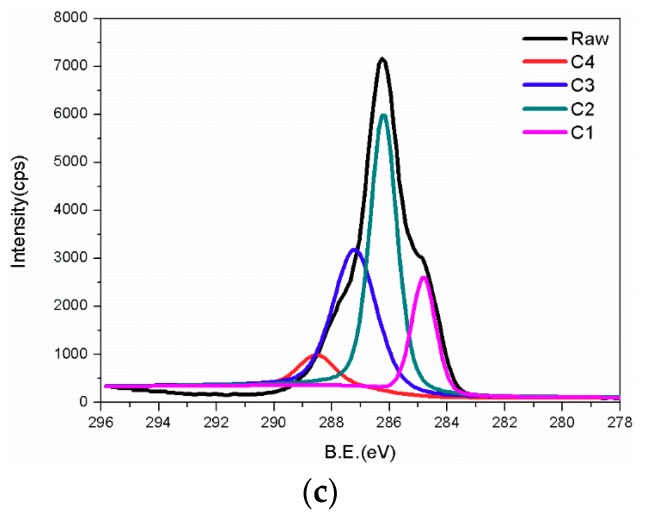
Accurate spectra of C1s related to P1, P2, and P3) (“(**a**)”, “(**b**)“, ”(**c**)“ are attached to P1, P2, and P3. ”Raw“ is original spectrum of C1s. ”C1, C2, C3, C4“ is type of bond connected to carbon and divided by ”Raw“.).

XPS analysis aims to identify types of bonds in cellulose and calculate relative content of each bond. Figure 8 lists full spectra of three types of treated pulp fiber. Figure 9 lists C1s accurate spectra for three types of treated pulp fiber.

Two principal peaks around 533 and 286 eV correspond to oxygen and carbon, respectively, in the spectrum of unmodified cellulose. A small peak at 1072 eV is related to sodium, because sodium hydroxide was adsorbed on the hydroxyl of cellulose during alkali treatment. However, only oxygen and carbon were detected in modified cellulose. Sodium hydroxide was removed from cellulose chain due to the penetration of pivaloyl chloride and solvent added during chemical modification. One big advantage of XPS analysis is access to specific type of bond via identifying an accurate element spectrum. As illustrated by Figure 9, the C1s signal that was collected from three kinds of treated cellulose was divided into four sub-peaks attached to four types of bond. Based on these peaks at 284.9, 286.4, 287.7, and 288.9 eV, carbon can be identified as C1 (C–C/C–H), C2 (C–O), C3 (O–C–O/C=O), and C4 (O–C=O), respectively [23]: 

(i). The C1 peak at 284.9 eV usually represents alkane-type carbon atoms. Cellulose chain includes this type of carbon partly and some of them exist in impurities, like lignin, extractives, and fatty acids [24].

(ii). The C2 peak at 286.4 eV is the contribution of the presence of ether groups (C–O–C). C–OH of the unmodified glucose ring is also responsible for this signal. Most of carbon atoms on cellulose are concerned with this peak [25].

(iii). The C3 peak at 287.7 eV is mainly attributed to carbonyl groups produced by oxidation. Another fractional contribution comes from a few carbon atoms of the AGU unit in cellulose. Here, the change of this signal is connected with the degree of oxidation.

(iv). The C4 peak at 288.9 eV is derived from ester groups, which is absent in unmodified cellulose. Its presence in P1 might be related to formation of carboxyl via oxidation in the alkali condition.

Here, the content of four types of carbon in P1 is set as reference and variation of content of C3 reflects total of carbonyl from surface oxidation and that within newly formed ester during modification. For content of C4, its change related to substitution determines degree of grafting. In this way, the ratio of oxidation to grafting can be roughly measured with equations 1–3, as presented below:

NG = [C4% − C4%(P1)] × TNC(1)

NO = [C3% − C3%(P1)] × TNC − NG(2)

R(O:G) = NO/NG = [C3% − C3%(P1)]/[C4% − C4%(P1)] − 1(3)

where NG and NO are the number of grafting and number of oxidation. TNC means total number of carbon. C4% and C3% are percentage of C4, C3 in cellulose. (P1) attached refers to specific percentage of C4 or C3 inside P1. R(O:G) is the ratio of oxidation to grafting.

In Table 3, the relative content of characteristic bond is obtained by calculating the peak area for a proportion of respective types of carbon within the whole C1s signal. A comparison for changes of four featured groups in cellulose before and after modification helps to achieve more evidence regarding chemical treatment.

**Figure 10:**
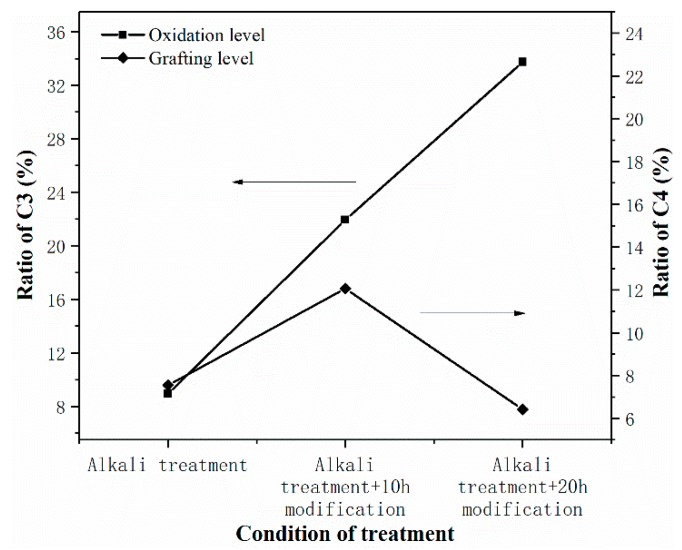
Changing ratio of C3 and C4 related to oxidation level and grafting level under varied conditions of treatment.

Figure 10 shows a schematic diagram about oxidation and grafting level under varied conditions based on the proportion of C3 and C4 in cellulose.

It is worth noticing that the degree of oxidation increases with the extension of modification time, which is indicated by the increase in C3 from P1 to P3. It results from alkaline condition induced by rid of sodium hydroxide that was adsorbed on cellulose during alkali immersion. The hydroxyl groups of cellulose tend to be oxidated in alkaline environment over 40 °C. In addition, the proportion of C4 in P2 reaches up to 12%, but it drops when prolonging reaction time to 20 h. This can be ascribed to the hydrolysis of ester. What is found in C4 also applies to the change of C1. Tertiary butyl of pivaloyl chloride grafted on cellulose accounts for the increase in C1 from P1 to P2. After the grafting reaction came to a certain degree hydrolysis became the principal reaction in last period of T3. Therefore, the reduction of tertiary butyl owing to hydrolysed ester leads to a decrease in C1 of P3.

In general, the degree of grafting is not high. For the ideal or higher degree of substitution, one had to add plenty of reagent, which produced considerable waste and caused difficulty in recycling. When compared to T3, T2 modification maintains a balance between esterification and oxidation. Great interfacial adhesion in P2 and ABS implies the effectiveness of T2 modification. It means that moderate surface oxidation on cellulose and slight grafting with branched tertiary butyl structure has synergic effects on improving the interface. For questing mechanism of interfacial reinforcement interaction among components needs to be examined. Herein, further investigation in compatibility between fiber and ABS was conducted.

### 3.5. DMA Test

**Figure 11:**
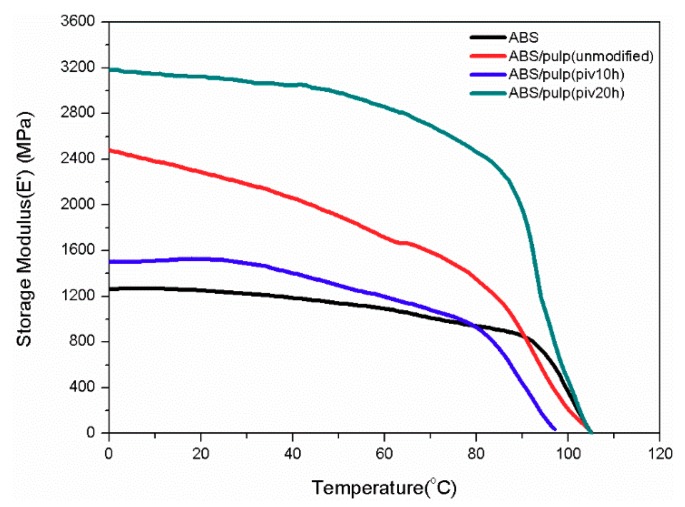
Varying curves of storage modulus with temperature at 1 Hz. (“ABS/pulp(unmodified)”, “ABS/pulp(piv10h)”, and “ABS/pulp(piv20h)” correspond to S1, S2, and S3, respectively).

**Figure 12:**
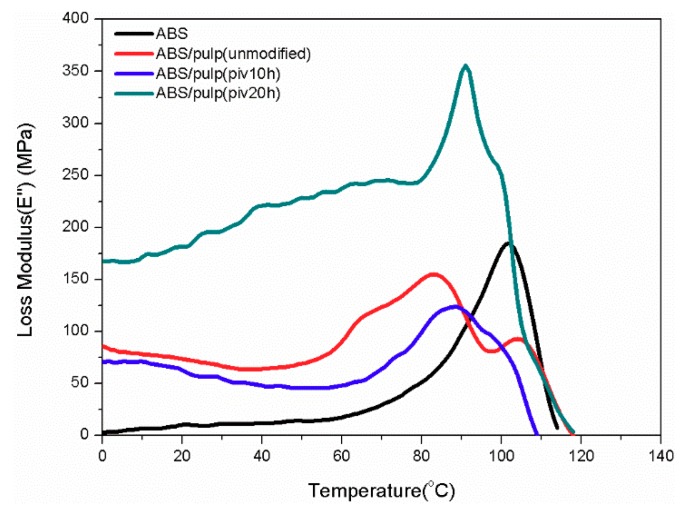
Varying curves of loss modulus with temperature at 1 Hz.

**Figure 13:**
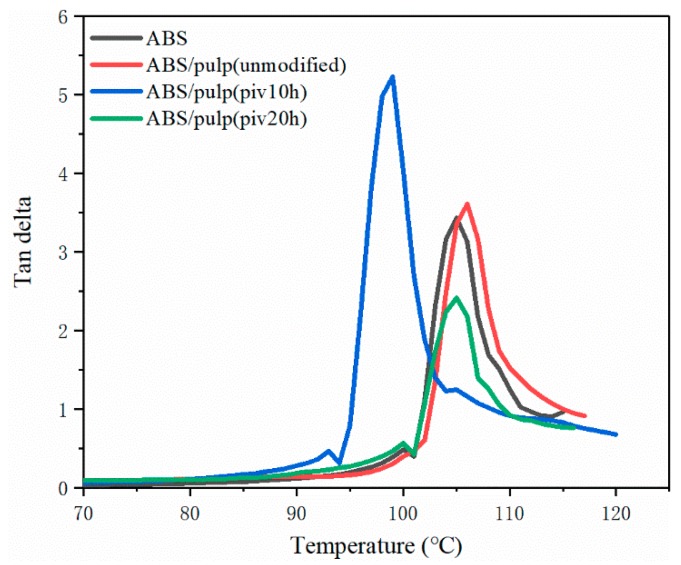
Varying curves of tan delta with temperature at 1 Hz.

**Figure 14:**
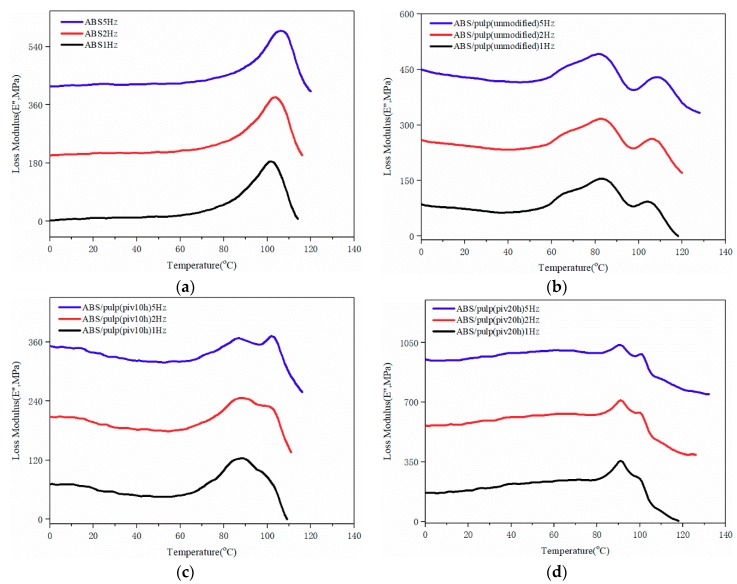
Varying curves of loss modulus of four samples under 1,2,5 Hz. (“(**a**)”, “(**b**)”, “(**c**)”, “(**d**)” represent pure ABS, S1, S2, and S3 respectively).

DMA was used for thermal and mechanical properties of composite under dynamic force and the changed frequency. Glass transition temperature (*T*_g_) and loss performance can be obtained from the DMA test. By comparing the trends of mechanical behaviour internal relations of composites are evaluated. Figure 11, Figure 12, Figure 13 and Figure 14 manifest the loss, storage modulus, and tan delta under variant temperature.

Figure 11 reveals that the storage modulus of all the samples steeply descends as temperature rises over 80 °C. This can be ascribed to the sliding of molecular segments when chaotic motion accelerates around the point of *T*_g_. Based on law for intersection of tangents *T*_g_ of several samples are found. Glass transition of S2 starts at 82.7 °C, which is the earliest point among four samples. Pure ABS reaches its *T*_g_ at 94.1 °C after the arrival of other three samples. In addition, S1 and S3 have *T*_g_ of 84.7 and 90 °C, respectively. The potential reason behind the difference in glass transition point is varying compatibility between two components in composites that restricts flexible displacement. It suggests the easy conformational rearrangement and increased free volume in molecular chains of S2 [26]. Moreover, Figure 12 compared the trends of loss modulus against changing temperature in this four samples for characterizing compatibility. As inferred by black curve, pure ABS has a single loss peak at 101.9 °C. Nevertheless, there are double isolated loss peaks at 83.1 and 104 °C in S1 as a result of poor compatibility. On the contrary, these two loss peaks combine into a wide peak, ranging from 80 °C to nearly 100 °C in S2. It proves the good compatibility between fiber and ABS [27], which is consistent with SEM observation. The loss curve of S3 manifests double adjacent loss peaks at 91 °C and 100 °C, owing to a partially miscible system. Its insufficient tensile strength might be due to the small size of P3, which probably leads to aggregation of fibrils and stress concentration [28]. This inference is also supported by SEM pictures of S3. It is noticeable that loss peak at about 100 °C corresponds to ABS, while the maximum loss modulus of pulp fiber appears at around 80 °C. In well miscible composite strong binding inhibits performance of single constituent in molecular motion, thus initiating the integrated thermal mechanical property. The best exemplification is found in S2, whose centralized loss peak starts from 80 °C of pulp fiber to 100 °C of ABS. Otherwise, two separate loss peaks emerge and it demonstrates weak interaction among constituents in the composite. A similar study in the thermo mechanical behaviour of better compatibilized PLA/ABS composite also attests this result [29]. Meanwhile, the variation of tangent delta in Figure 13 also confirms interface improvement of S2. The loss factor of S2 reaches maximum point, which is the highest level among four samples, before the temperature rises to 100 °C. It indicates dissipated energy is far greater than stored energy inside S2. This can be ascribed to fine interfacial adhesion that tightly binds phases and facilitates energy transfer from ABS to pulp fiber. However, the loss factor of S1 and S3 resembles that of neat ABS because poor interfacial binding makes it difficult for pulp fiber to dissipate the energy and loss performance of ABS occupies the dominant place in composite. For S3, its peak loss factor is lower than neat ABS. The reason might be that damage to morphology of pulp fiber that is initiated by undue oxidation leads to incomplete energy dissipation. Besides, loss behaviours under higher frequency are analysed in order to seek varying patterns of interfacial binding further. As to pure ABS in Figure 14a, the sole loss peak shifts from 101.9 °C at 1 Hz to 106 °C at 5 Hz, but width of peak remains constant. From the perspective of effects on segment motion, higher frequency means a shorter action time that is matched with higher temperature. Different from (a), Figure 14b depicts that the gap between two loss peaks expands as frequency rises. The peak related to ABS moves to higher temperature, while the peak for pulp fiber appears earlier. The maximum loss of fiber is brought forward, because it is more difficult for rigid cellulose chain to respond under higher frequency. For S2, there exists an obvious separation of wide peak into double isolated peaks when increasing the frequency to 5 Hz. This implies that insufficient binding leads to worse compatibility. Owing to higher frequency, previous strong interaction between two components is weakened. Consequently, the integrated structure is damaged and the composite exhibits separate mechanical loss. When compared to S2, the expanding gap between two loss peaks in S3 is not remarkable when applied to higher frequency. The loss peak at 100 °C takes a shift to higher temperature while the peak at 91 °C almost remains steady. Intensity of peak related to ABS increases, but the peak intensity of pulp fiber decreases. This varying intensity reflects the change of loss modulus, which indicates the flexibility of constituent. Therefore, softness can be observed in ABS and stiffness is gradually detected in pulp fiber with incremental frequency. It infers that their segment motion lags behind frequency. What is found is more evident in pulp fiber of S3 than in that of S1 and S2. Hindered chaotic motion that is induced by a reduction in flexibility of cellulose chain is responsible for this result. Slight degradation might arise and polymerization degree of cellulose declines subsequently, followed by poor flexibility of chain, while considering the degree of oxidation in P3.

### 3.6. Structure Design for Composite

Accordingly, T2 modification for pulp fiber strengthens interaction among molecules in composite and compatibility between fiber and ABS is improved. Apart from modification the addition of PAM in all three composites cannot be overlooked. Notably seen in pre-experiment, the apparent performance was significantly optimized when adding five percent PAM in pulp fiber/ABS composite with 1:1 content ratio. The rough surface of sample without PAM was transformed into smooth layer, which was an indicator that PAM played an important role in promoting miscibility. Inspired from interlayer adhesion in three-dimensional (3D) printing with increased diffusion and entanglement of chains [30], interface reinforcement of ABS composite is achieved, along with the process of melt extrusion. An internal structure model was built for understanding the interaction among three elements of composite.

**Figure 15:**
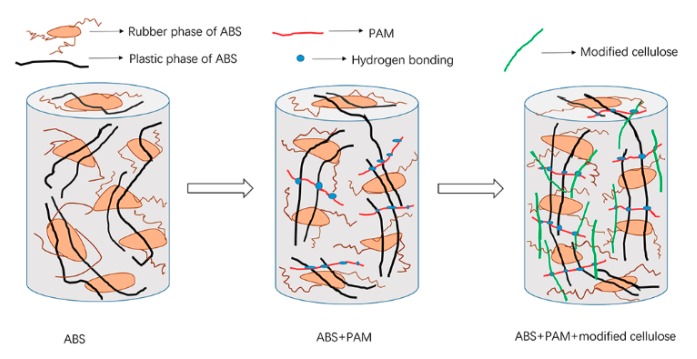
Three-element reinforcing structure of composite.

As illustrated in Figure 15, brown circles with curved segments around represent soft rubber phase of ABS, while black lines simulate rigid plastic chains. When PAM is added, hydrogen bonding between NH of peptide unit and cyano group of polyacrylonitrile blocked in copolymer of plastic chain is formed. Furthermore, on one hand, carbonyl group of modified cellulose thanks to oxidation of hydroxyl receives hydrogen bonding from PAM via oxygen atom. On the other hand, branch shaped tertiary butyl grafted on cellulose fiber is entangled with a flexible chain of polybutadiene rubber by van der Waals force. PAM bridges cellulose fiber and ABS. The formation of interlinked three-element structure completes interfacial reinforcement and, thus, increases the efficiency of load transfer. The mechanical properties are upgraded as a result.

Additional information about morphology and sketch map of interlinked structure can be obtained from Figures S1 and S2 in Supplementary Materials.

## 4. Conclusions

In our work, a kind of natural fiber reinforced ABS composite is prepared by melt compounding. Functionalized modification on cellulose fiber, including moderate surface oxidation and slight grafting with tertiary butyl, was carried out. A favourable procedure starting from alkali immersion for one hour and ending up with ten-hour pivaloyl chloride reaction was adopted by comparing three types of treatment to cellulose fiber. It was an economic modification marked by limited modifier, whose mass ratio to pulp fiber only came to 1:1.6 as compared to Agustin’s method [8], and less side products. With the addition of 5 wt % PAM apparent performance of composite as well as miscibility of fiber and ABS was improved due to the formation of interlinked three-element structure.

This project is centered on interfacial reinforcement in natural fiber-based ABS composite. The results of chemical treatment revealed inhibited grafting reaction, but mild controllable surface oxidation. Microscopic morphology and thermal mechanical performance of the composite verify that surface oxidation and slight grafting have synergic effects on improving compatibility. Ascribed to great miscibility, the interfaces between fiber and ABS are finally enhanced. Overall, interfacial optimization was realized by hydrogen bonding, owing to stepwise treatment on pulp fiber with low-volume and recycled reagents in expedient time. Melt compounding via twin screw extruder and injection moulding ensured continuous processing. High filling natural fiber reinforced ABS biocomposite is desired to be utilized based on this study.

## Figures and Tables

**Table 1:** Comparison of three types of treatment in reaction condition.

Modification Type	Alkali Treatment (40 °C, 1 h)	Conditions of Modification				
	Concentration (wt %)	Solvent	Piv:Pulp *	Piv:Pyridine *	Temperature (°C)	Time (h)
1	16%	/	/	/	/	/
2	16%	NMP	1:1.6	0.4:1	80	10
3	16%	NMP	0.9:1	1.1:1	80	20

**Note:** “1,2,3” are labels of treatment. “/” means non-existent item and “*” represents mass ratio. “Piv” is abbreviation of pivaloyl chloride.

**Table 2:** Tensile strength and tensile modulus of neat acrylonitrile-butadiene-styrene (ABS) and ABS composites.

Sample	Tensile Strength (MPa)	Tensile Modulus (MPa)
Neat ABS	39.3 (0.37)	1574
S1	45.8 (0.76)	2567
S2	58.1 (0.85)	2515
S3	43.1 (0.48)	2640

**Note:** Value in brackets is standard deviation.

**Table 3:** Distribution of C1, C2, C3, and C4 in cellulose.

	Proportion of Various Types of Carbon (%)			
	C1	C2	C3	C4
P1	26	57	9	8
P2	31	35	22	12
P3	17	43	34	6

## Supplementary Materials

The supplementary materials are available online at https://www.mdpi.com/2073-4360/11/12/2048/s1.
