# Supplementary material for: Interface Reinforcement of Pulp Fiber Based ABS Composite with Hydrogen Bonding Initiated Interlinked Structure via Alkaline Oxidation and tert-Butyl Grafting on Cellulose
Source: Polymers (Basel). 2019 Dec 10;11(12):2048. doi: 10.3390/polym11122048 (PMC6960529; doi:10.3390/polym11122048)
Supplement: Supplementary file 1 [file polymers-11-02048-s001.pdf]

Electronic Supplementary Information (ESI)

Interface reinforcement of pulp fiber based ABS bio-composite via functionalized modification-induced interlinked structure

Qinrui Zhu and Dagang Li*

Address: College of Material Science and Engineering, Nanjing Forestry University, No. 159, Long Pan Road, Nanjing, 210037, China

E-mail: zhuqinrui1996@njfu.edu.cn

njfuldg@163.com

This Supporting Information contains two figures.

Results and Discussion

Evidence for interlinked structure

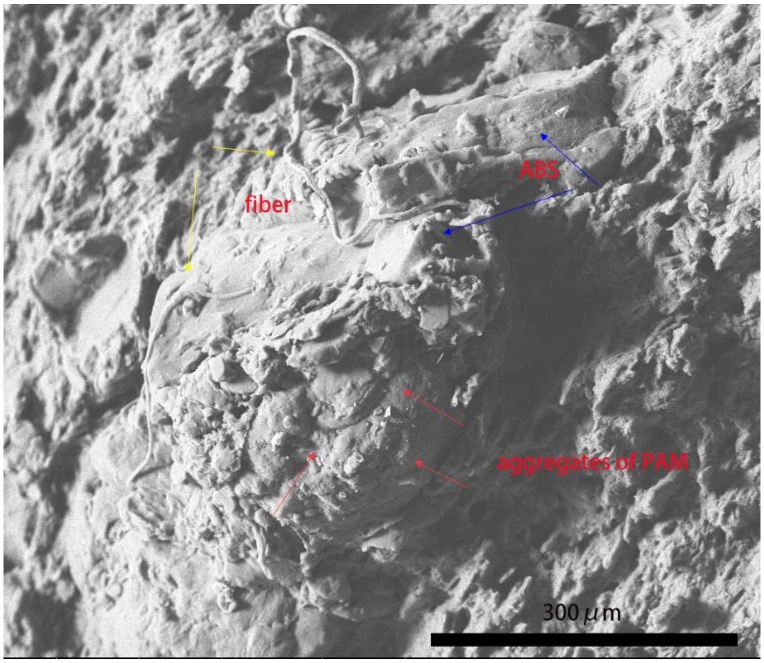

Figure S1. SEM observation for morphology of extracted pulp fiber, ABS and PAM at a scale bar of 300 μm after fracture of S3.

Due to degradation in P3, its mechanical performance gets worse than that of P2. When applied to tensile stress, pulp fiber combined with residue matrix are pulled out. However, an interlinked structure can be found above. As shown in figure S1, extracted fiber is buried in ABS on one side and the other side of fiber is coated with aggregates of PAM. Obviously, fractional pieces of PAM form into granules and adhere with ABS grains. Pulp fiber penetrates through the bulk of ABS/PAM mixture. There exists great interaction among

fiber, ABS and PAM. In order to depict this interlinked three-element structure, a sketch map about intermolecular force is presented below.

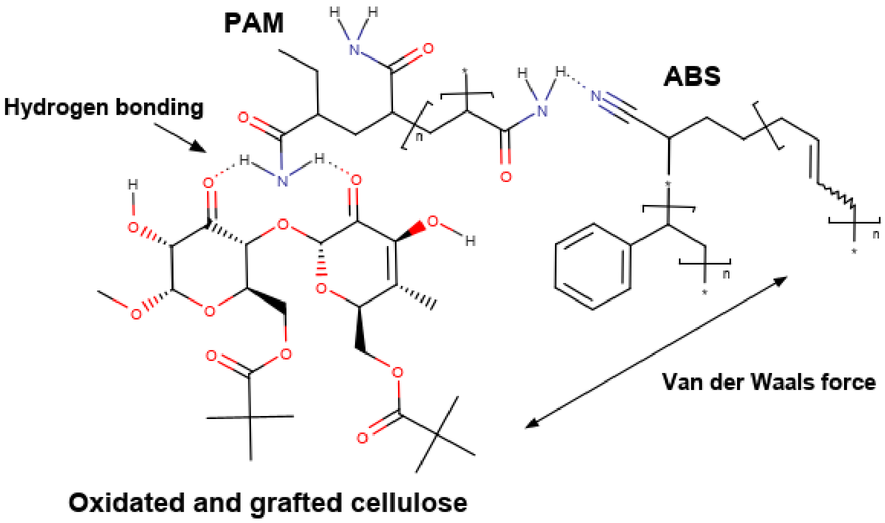

Figure S2. Molecular sketch map of interlinked three-element structure.

During modification part of secondary hydroxyl undergo oxidation followed by formation of olefinic bonds conjugated with carbonyl. Branch sized tertiary butyl tend to substitute primary hydroxyl and form ester functions. Carbonyl groups on cellulose play a role as receptor of hydrogen bonding supplied from PAM. Meanwhile, hydrogen bonding links ABS to PAM as well. Besides, there is interaction between tertiary butyl and polybutadiene phase of ABS via Van der Waals force owing to similar polarity. By intermolecular force this interlinked three-element structure is built.
